# Quantifying the invasion and migration ability of cancer cells with a 3D Matrigel drop invasion assay

**DOI:** 10.1093/biomethods/bpab014

**Published:** 2021-07-21

**Authors:** Merve Aslan, En-Chi Hsu, Shiqin Liu, Tanya Stoyanova

**Affiliations:** Department of Radiology, Canary Center at Stanford for Cancer Early Detection, Stanford University, 3155 Porter Drive. Palo Alto, CA 94304, USA

**Keywords:** cancer, migration, metastasis

## Abstract

Metastasis is the main cause of cancer-associated morbidity which will account for ∼ 600,000 deaths in the USA in 2021. Defining new mechanisms that drive cancer metastasis is vital for developing new therapeutic strategies and improving clinical outcomes for cancer patients. Herein, we describe a recently established 3D Matrigel drop invasion assay to measure cancer cell invasion and migration capability *in vitro*. This assay is a versatile and simple tool to test the ability of cells to invade and migrate, test the functional role of genes of interest in cell invasion and migration, analyze the localization of the target proteins at the cell invasion edge *in situ*, and screen drug effects on cancer cell invasion and migration.

## Introduction

Metastasis is one of the hallmarks of cancer and remains the main contributor to cancer associated deaths [1–4]. Cancer cells break away from the primary tumor by local invasion, penetrate to the circulatory system, and further escape from the blood vessels to colonize into distant sites [2, 5–9]. After the detachment from the primary tumors, metastatic cells start synthesizing matrix metalloproteinases to invade through the extracellular matrix by digesting the basal membrane [10, 11]. Extrapolating the *in vivo* environment to *in vitro* to be able to measure cancer cell migration and invasion abilities is crucial [12, 13]. Cancer cell invasion abilities have been commonly investigated using Matrigel-coated Boyden chambers and conditioned media as a chemoattractant [14–18]. The cancer cell invasion behaviors that were identified by modified Boyden chamber assays have been confirmed with a number of *in vivo* studies underlying the efficacy of the technique. Measuring cancer cell invasion by generating spheroids and suspending them into a gel or on top of a gel has also been studied extensively [19–24]. However, these techniques are suitable to analyze cell invasion only at the end point.

Herein, we provide a detailed protocol of a novel technique to test the invasion and migration ability of cells in 3D platform *in vitro*. Cancer cells are mixed with Matrigel scaffold [25, 26]. The mixture of cells and Matrigel is plated as a single drop in a well of a 24-well plate and supplemented with media which allows for monitoring the invasion and migration ability of the cells out of the Matrigel drop over a period of time (Table 1). More importantly, this assay allows for live cells imaging during the invasion process or fixed cells staining for further analyses at end point (Table 1). We have utilized this assay to analyze various drug treatment effects on cancer cell invasiveness and to test the invasion ability of cancer cells that are genetically manipulated [27–30]. This method was used to test the effect of Poly (ADP-ribose) polymerase (PARP) inhibitors (Olaparib and Talazoparib), Ciprofloxacin, ferroptosis inhibitors (Erastin and RSL3), and gamma secretase inhibitors (DAPT and RO4929097), alone or in combination with second generation anti-androgens Enzalutamide and Abiraterone on migration and invasion abilities of different prostate cancer cell lines [27–30]. Moreover, 3D Matrigel drop assay was performed to test the Trop2 oncogene overexpression effect in migration and invasion capability of prostate cancer cell line LNCaP [27]. In summary, the 3D Matrigel drop invasion assay is a robust, easy, and reproducible technique to assess cell invasion and migration upon genetic alterations and to monitor drug treatment effects on cancer cell invasion and migration (Table 1).

**Table 1:** Advantages of the 3D Matrigel drop invasion assay

1. Monitoring the migration and invasion throughout the experiment and at the experimental end point
2. Study the localization of proteins of interest at the invasion edge *in situ*
3. Test and monitor drug treatment effects on migration and invasion abilities of cancer cells over time
4. Analyze the functional role of genes of interest on migration and invasion of cells

## Materials and methods

### Cell lines and reagents

MCF7 (ATCC^®^ HTB-22), MDA-MB-231 (ATCC^®^ HTB-26), MDA-MB-468 (ATCC^®^ HTB-132), and HCC1806 (ATCC^®^ CRL-2335) are used. Breast cancer cell lines are authenticated through the Genetica cell line testing. LNCaP (ATCC^®^ CRL-1740), DU145 (ATCC^®^ HTB-81), PC3 (ATCC^®^ CRL-1435), and 22RV1 (ATCC^®^ CRL-2505) cells are purchased from ATCC and are authenticated through the Stanford Functional Genomics Facility. All the cell lines are inspected for mycoplasma with Mycoalert Detection Kit (#NC9719283, Lonza). Cells are cultured in RPMI 1640 (Thermo Fisher Scientific) media supplemented with 10% FBS (#F0926, Sigma-Aldrich) and 1% penicillin/streptomycin (#PSL01-6X100ML, Caisson Labs), and 1% glutamax (#35-050-061, Gibco) and kept in 37°C incubator with 5% CO_2_. Cells are dissociated by warm 0.25% Trypsin/EDTA (#T4174-100ML, Sigma-Aldrich) solution.

### Cell preparation

Cells are counted for each drop with a cell counter (Countess II Automated Cell Counter, Invitrogen) and the required number of cells (5 × 10^4^/drop) are added into a 1.5 ml Eppendorf tube in the conditioned media for the cells. The tubes are centrifuged at 1000*g* for 5 min at 4°C. The supernatant media is removed with gentle pipetting avoiding losing any cell from the cell pellet.

### Generating the Matrigel drops

**Figure 1:**
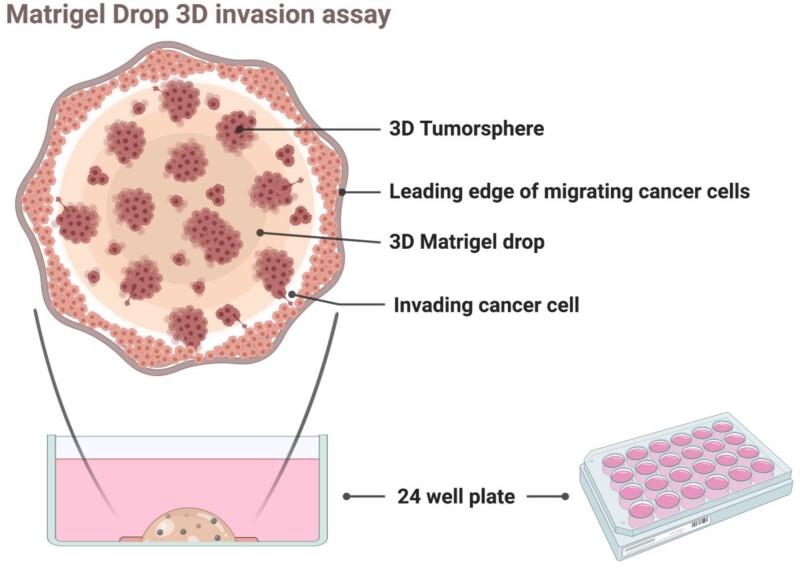
3D Matrigel drop invasion assay. The cartoon image describes the 3D Matrigel drop. The enlarged view of the assay demonstrates the 3D tumorsphere structure and the invaded cells out of the Matrigel. The image was generated using BioRender.

Matrigel (#356235, Corning) is thawed on a shaker at 4°C for 2 h. Matrigel is added onto the cell pellet in an Eppendorf tube and mixed gently avoiding generation of bubbles. 10 µl of Matrigel is used per drop and these steps are performed on ice. Cells that are mixed with cold Matrigel are gently pipetted into the middle of a well in a 24-well plate into a drop-like shape. For seeding the cells as a drop-like shape, the pipet has to be placed exactly in 90° angle in the middle of the well and the edge of the tip could touch to the plate gently. The cells and Matrigel mixture is pipetted slowly without generating any bubbles in the droplets. The cells and Matrigel mixture should be prepared using extra volumes to account for pipetting errors (for triplicate, a mixture for four drops should be prepared: 2 × 10^5^ cells should be mixed with 40 µl of Matrigel). The Matrigel drops are solidified in a 37°C incubator with 5% CO_2_ injection for 20 min. The Matrigel drop solidification should not exceed 20 minutes to avoid drying out the cells. After the drops are solidified, 2 ml of cell type specific media is added into each well. The media should be added to the wall of the well to avoid destruction of the 3D Matrigel drop structure. The cells are kept in culture for 6 days and the media is changed at Day 3. The assay is described in Fig. 1.

### Quantification of the image data

**Figure 2:**
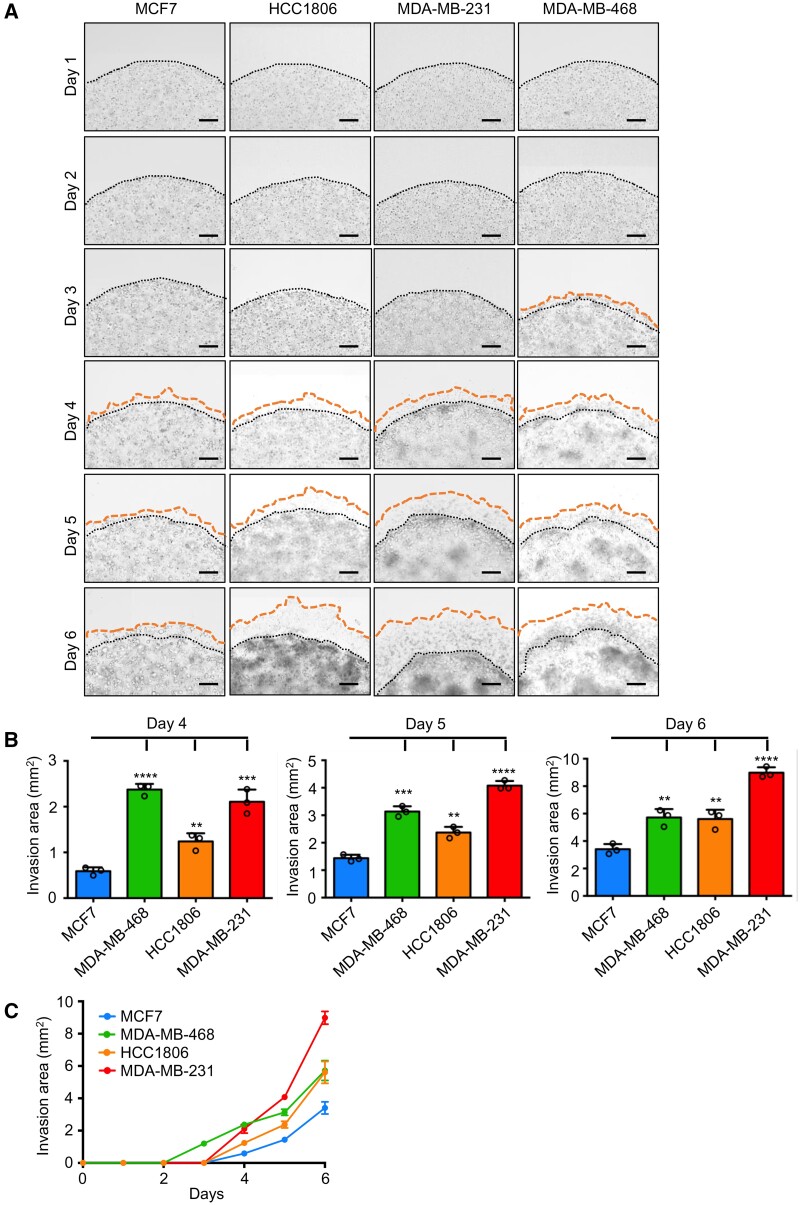
Invasion and migration ability measurement of breast cancer cell lines with 3D Matrigel Drop invasion assay. (A) Matrigel drop brightfield images of breast cancer cells MCF7, HCC1806, MDA-MB-231, and MDA-MB-468 at Days 1, 2, 3, 4, 5, and 6. A mixture of 5 × 10^4^ cells and with 100% Matrigel was seeded as a drop-like shape in triplicates and grown for 6 days in culture. The cells were imaged every day. The borders of the Matrigel drop were labeled with black dotted lines and the borders of the migrated cells were marked with orange dotted lines. Scale bars represent 200 µm. (B) The plots illustrating the quantified invasion area of each breast cancer cells at Days 4, 5, and 6. (C) Invasion and migration area measurements of each breast cancer cell line in the 3D Matrigel drop invasion assay over 6 days. Error bars represent standard deviation (SD). ^**^P < 0.01, ^***^P < 0.001, and ^****^P < 0.0001 are derived from two-tailed Student’s *t* test.

**Figure 3:**
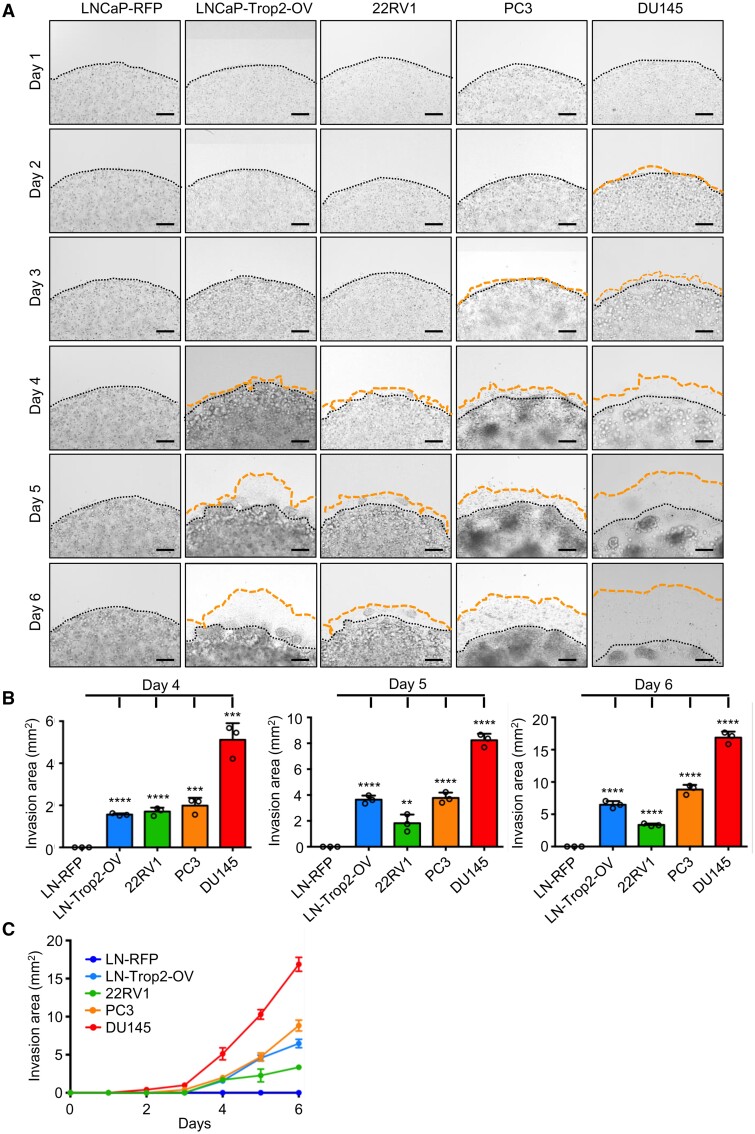
Invasion and migration ability quantification of prostate cancer cell lines with 3D Matrigel Drop invasion assay. (A) Daily images of prostate cancer cell lines LNCaP-RFP, LNCaP-Trop2-OV, 22RV1, PC3, and DU145 that were mixed with Matrigel and seeded as a drop-like shape in a well of a 24-well plate. Black dotted lines represent the edge of the Matrigel drop and orange dotted lines demonstrate the edge of the invaded cells out of the Matrigel. Scale bars represent 200 µm. (B) The plots displaying the calculated invasion area of the prostate cancer cells at Days 4, 5, and 6. (C) Prostate cancer cells invasion and migration out of the Matrigel drop over six days. Error bars represent SD. ^**^P < 0.01, ^***^P < 0.001, and ^****^P < 0.0001 are derived from two-tailed Student’s *t* test.

Cells can be imaged every day with a light microscope or Celigo S Imaging Cytometer (200-BFFL-S). In the presented experiments here, the migrating edge outside the Matrigel drop was measured with ImageJ. A line was drawn with the freehand tool of ImageJ to measure the entire area with the Matrigel drop and the migrated cells. Another line was drawn to measure just the Matrigel drop area itself. The Matrigel drop area was further subtracted from the total area that was covered with the migrated cells. At last, the pixel quantification of the area was converted to square millimeter with setting the scale in ImageJ. Student’s *t* test was used to perform two-tailed analysis in Figs 2B and 3B.

### Immunofluorescence staining

Cells are washed with 1x Phosphate-buffered saline (PBS) and fixed with cold 1:1 acetone (#A929-1, Thermo Fisher Scientific)/methanol (#A412P-4, Thermo Fisher Scientific) for 20 min at −20°C. Cells are then blocked in 5% bovine serum albumin (BSA, #BP9706100, Thermo Fisher Scientific) overnight at 4°C. Before staining, the cells are scanned through Celigo S Imaging Cytometer in order to verify the absence of any immunofluorescence signal. E-cadherin (#3195S, Cell Signaling Technology) and Trop2 antibodies (sc-376746, Santa Cruz) are added in 1:200 dilution in PBS onto the cells for overnight incubation at 4°C. Cells are further washed three times for 5 min with 1xPBS and secondary antibodies anti-rabbit Alexa flour 594 (#NC0208612, Fisher Scientific) and anti-mouse Alexa flour 594 are applied for 2 hours at room temperature. After three times washing with 1xPBS for 5 min each, cells are mounted with 20% DAPI mounting solution (#0100-20, SouthernBiotech) and 20% glycerol (#BP229-1, Thermo Fisher Scientific) in 1xPBS.

## Results

### Quantifying cancer cells invasion and migration ability with 3D Matrigel drop invasion assay

We first measured the invasion and migration ability of different cancer cell lines with 3D Matrigel drop invasion assay (Figs 2 and 3). The Matrigel-embedded cells were imaged every day. Breast cancer cells, MDA-MB-468 cells started invading through the Matrigel at Day 3, whereas MCF7, MDA-MB-231, and HCC1806 cells invasion was initiated at Day 4 (Fig. 2A and B). MCF7 cells have demonstrated less invasive phenotype whereas MDA-MB-231 was the most aggressive cell line in terms of invasion and migration (Fig. 2C). We identified the invasion pattern of each cell line for 6 days with 3D Matrigel drop invasion assay (Fig. 2C). In the context of prostate cancer, DU145 and PC3 displayed the highest invasive and migration ability among the prostate cancer cell lines analyzed here and invaded the Matrigel drop starting at Days 2 and 3 (Fig. 3A and B). This assay provides the opportunity to monitor the invasion and migration of cancer cells not only at the experimental point but also throughout the 6-day experiment. The results indicate that depending on the cell type 4–6 days culturing is the most optimal timeline for this assay to evaluate cell invasion and migration.

### Measuring the invasion and migration ability upon genetic alterations in cancer cells using 3D Matrigel drop invasion assay

**Figure 4:**
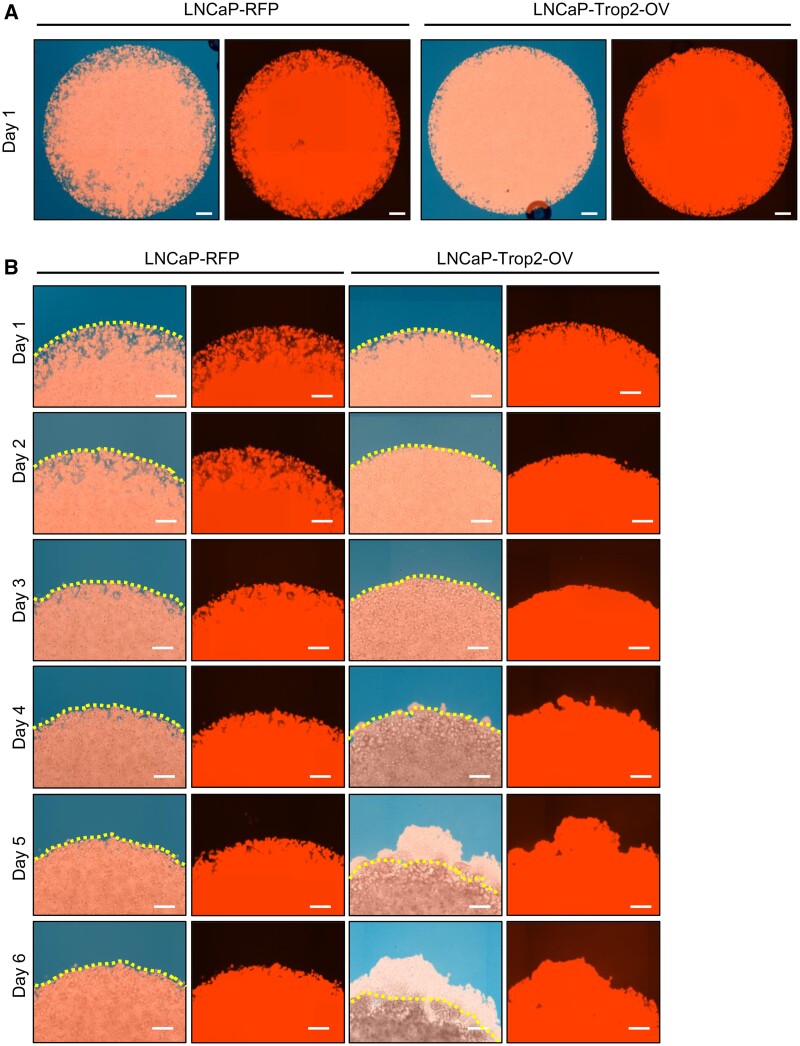
Comparison of genetically engineered cell lines invasion and migration abilities using 3D Matrigel drop invasion assay. (A) The brightfield—RFP merged and RFP only images of the whole Matrigel drop seeded with LNCaP-RFP and LNCaP-Trop2-OV prostate cancer cells at Day 1. The scale bars represent 400 µm. (B) The merged and RFP only images of LNCaP-RFP and LNCaP-Trop2-OV cells in Matrigel drop at higher magnification at Days 1, 2, 3, 4, 5, and 6. Dotted lines in merged images represent the border of the Matrigel drop. Scale bars represent 200 µm.

Next, we compared the invasion and migration ability of genetically engineered cancer cells to parental cells with 3D Matrigel drop invasion assay (Figs 3 and 4). LNCaP cells were derived from lymph node metastasis of a prostate cancer patient [31]. Trop2 is a well-known oncogene that is a cell surface glycoprotein and Trop2 overexpression in LNCaP cells causes metastatic phenotypes in prostate cancer [27]. Since LNCaP cells do not migrate or invade through transwell chambers, we have used the 3D Matrigel drop invasion assay to assess the invasion and migration ability of LNCaP and Trop2 overexpressing LNCaP cells [27]. The generation of stable LNCaP cells that are either expressing red fluorescence protein (RFP) (LNCaP-RFP) or Trop2 and RFP (LNCaP-Trop2-OV) was described previously [27]. LNCaP-Trop2-OV cells invasion and migration were significantly increased compared to the LNCaP-RFP cells (Fig. 3B and C). These results demonstrate that the 3D Matrigel drop invasion assay gives the opportunity to evaluate the invasion and migration capacity of cells upon genetic alterations and test the role of specific genes in invasion and migration.

### Fluorescent imaging of 3D Matrigel drop to assess invasion phenotype of the cancer cells

Fluorescent imaging of the 3D Matrigel drop allows for further assessment and visualization of migration and invasion abilities. Utilizing the assay with any fluorescent gene expressing cell line permits the fluorescent imaging to visualize cell invasion and migration. We have imaged the red fluorescent signal of the 3D Matrigel drop with LNCaP-RFP and LNCaP-Trop2-OV cells, both expressing RFP (Fig. 4). Either RFP or brightfield imaging enables the quantification of the invasion and migration capacity of the cells.

**Figure 5:**
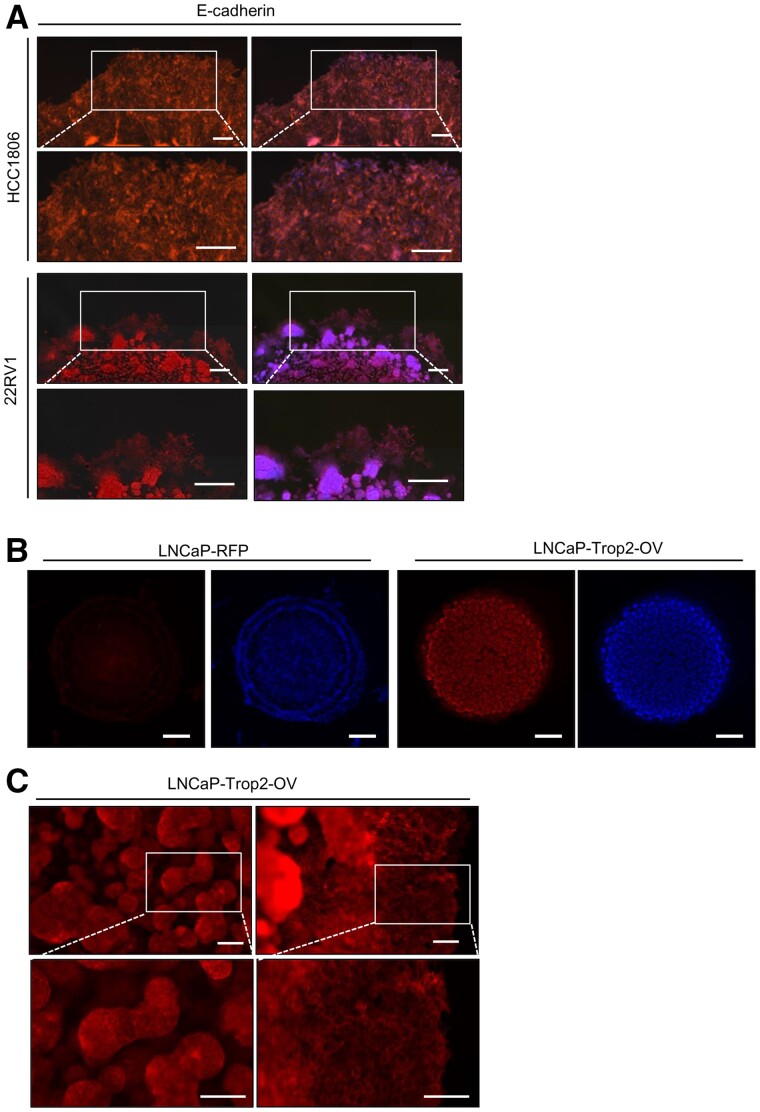
Immunofluorescent staining of 3D Matrigel drops. (A) Immunofluorescent staining images of HCC1806 and 22RV1 cells for E-cadherin after the 3D Matrigel drop invasion assay was performed. Cells were fixed in 1:1 acetone/methanol, blocked in 5% BSA, and stained for the target proteins. RFP and RFP-DAPI merged images are shown. Scale bars represent 200 µm. (B) The immunofluorescent staining pictures of LNCaP-RFP and LNCaP-Trop2-OV cells for Trop2 after the Matrigel Drop invasion assay. RFP and DAPI images of the whole drop. Scale bars represent 1 mm. (C) Higher magnification pictures of Trop2 stained tumorsphere inside the Matrigel drop (left) and invaded cells at the protruding edge (right) of LNCaP-Trop2-OV cells in 3D Matrigel drop invasion assay by Leica stereomicroscope. Scale bars represent 100 µm.

Besides the quantification of the invasion and migration ability of the cancer cells, the assay also allows for investigation of the cell phenotype by immunofluorescent staining for protein markers in the migrating edge *in situ*. In this study, we have performed immunofluorescent staining for E-cadherin to determine the migratory phenotype of the cells after the fixation (Fig. 5A). Both HCC1806 and 22RV1 cells expressed E-cadherin at the protruding edge (Fig. 5A). Moreover, we stained the LNCaP-RFP and LNCaP-Trop2-OV 3D Matrigel drops for Trop2 (Fig. 5B). The identified membrane pattern of Trop2 expression on the migrating cells or in 3D tumorsphere inside the 3D Matrigel drop demonstrated that this assay allows to investigate the localization of target proteins with subsequent imaging (Fig. 5B and C).

## Discussion

Generating new techniques to investigate the mechanisms of cancer metastasis *in vitro* will facilitate our understanding regarding mechanisms underlying cancer development and progression and identify new therapeutic strategies to halt cancer spreading. Here, we describe a novel cell and cancer invasion and migration assay that can precisely measure the invasion and migration abilities of cells. The cell invasion and migration ability are analyzed in a 3D environment by seeding a mixture of cells and Matrigel as a drop. This assay allows to visualize the cell invasion not only at the end point but also during the invasion and migration processes. Invasion through the Matrigel and migration ability of the cellsare monitored for 6 days by this method. Moreover, analysis of the cells for proteins of interest by immunofluorescent staining either in the tumorsphere Matrigel drop or in the protruding edge is another advantage of the assay. Our results suggest that the 3D Matrigel drop invasion and migration method is simple and versatile to study the invasion and migration ability of cells *in vitro*.

## Data availability

All the data that are presented are included in the article materials and further inquiries can be directed to the corresponding author.
